# Multidrug Resistant *Klebsiella pneumoniae* ST101 Clone Survival Chain From Inpatients to Hospital Effluent After Chlorine Treatment

**DOI:** 10.3389/fmicb.2020.610296

**Published:** 2021-01-11

**Authors:** Laura Ioana Popa, Irina Gheorghe, Ilda Czobor Barbu, Marius Surleac, Simona Paraschiv, Luminiţa Măruţescu, Marcela Popa, Graţiela Grădişteanu Pîrcălăbioru, Daniela Talapan, Mihai Niţă, Anca Streinu-Cercel, Adrian Streinu-Cercel, Dan Oţelea, Mariana Carmen Chifiriuc

**Affiliations:** ^1^Department of Botany and Microbiology, Faculty of Biology, University of Bucharest, Bucharest, Romania; ^2^Research Institute of the University of Bucharest, Bucharest, Romania; ^3^Department of Bioinformatics, National Institute of Research and Development for Biological Sciences, Bucharest, Romania; ^4^National Institute for Infectious Diseases “Matei Bals”, Bucharest, Romania; ^5^National Research and Development Institute for Industrial Ecology (ECOIND), Bucharest, Romania; ^6^Department II – Infectious Diseases, University of Medicine and Pharmacy “Carol Davila”, Bucharest, Romania

**Keywords:** wastewater treatment plant, MDR *Klebsiella pneumoniae*, whole-genome sequencing, hospital wastewater chlorine treatment, hospital sewage

## Abstract

In this paper we describe the transmission of a multi-drug resistant *Klebsiella pneumoniae* ST101 clone from hospital to wastewater and its persistence after chlorine treatment. Water samples from influents and effluents of the sewage tank of an infectious diseases hospital and clinical strains collected from the intra-hospital infections, during a period of 10 days prior to wastewater sampling were analyzed. Antibiotic resistant *K. pneumoniae* strains from wastewaters were recovered on selective media. Based on antibiotic susceptibility profiles and PCR analyses of antibiotic resistance (AR) genetic background, as well as whole-genome sequencing (Illumina MiSeq) and subsequent bioinformatic analyses, 11 ST101 *K. pneumoniae* strains isolated from hospital wastewater influent, wastewater effluent and clinical sector were identified as clonally related. The SNP and core genome analyses pointed out that five strains were found to be closely related (with ≤18 SNPs and identical cgMLST profile). The strains belonging to this clone harbored multiple acquired AR genes [*bla*_CTX–M–__15_, *bla*_OXA–__48_, *bla*_OXA–__1_, *bla*_SHV–__106_, *bla*_TEM–__150_, *aac(3)-IIa*, *aac(6′)-Ib-cr*, *oqx*A10, *oqx*B17, *fos*A, *cat*B3, *dfr*A14, *tet*(D)] and chromosomal mutations involved in AR (Δ*mgrB*, Δ*ompK35*, amino acid substitutions in GyrA Ser83Tyr, Asp87Asn, ParC Ser80Tyr). Twenty-nine virulence genes involved in iron acquisition, biofilm and pili formation, adherence, and the type six secretion system – T6SS-III were identified. Our study proves the transmission of MDR *K. pneumoniae* from hospital to the hospital effluent and its persistence after the chlorine treatment, raising the risk of surface water contamination and further dissemination to different components of the trophic chain, including humans.

## Introduction

Due to the worldwide use of antibiotics in the treatment of human and animal infectious diseases, but also in livestock and agriculture, a large amount of antibiotics of pharmaceutical origin are found in anthropic environments, such as sewage and wastewater treatment plants (WWTPs) and ends up being discharged in the natural environment (Kraemer et al., 2019; Zhang T. et al., 2019; Zhang Z. et al., 2019). The extensive use of antibiotics leads to the spread of antibiotic resistance (AR), which currently represents a major public health concern. AR rates are particularly high in acute care hospitals driven by selective pressure of antibiotic usage (Hocquet et al., 2016), hospitals being main ecological niches for the selection, accumulation, and spread of antibiotic resistant bacteria (ARB).

Water plays a crucial role in the spreading of AR, through inappropriate release of human and animal effluents in the surface waters through hospital wastewaters, WWTPs, aquaculture farms, surface, and groundwater. Hospital wastewaters are highly complex effluents, carrying a wide range of micro- and macropollutants, including antibiotic compounds, metabolized drugs, disinfectants, patient excrements, and microorganisms. The presence of ARB as well as of antibiotic residues, which could inhibit the growth of susceptible bacteria, are thereby increasing the population of resistant bacteria in the receiving water (Kaur et al., 2020; Rozman et al., 2020).

Hospitals generate a large amount of wastewater per day. The hospital effluents are loaded with pathogenic microorganisms, antibiotics, and other pharmaceutical or toxic substances, which are only partially removed during wastewater treatments, driving the pollution of the natural environments, including selection, and dissemination of AR (Kummerer et al., 2000; Kim and Aga, 2007; Alrhmoun et al., 2014; Laffite et al., 2016). Antibiotic pollutants, as well as heavy metals and even chlorination, could increase the general rates of mutation, recombination, and lateral gene transfer, thus recruiting more genes into the resistome and mobilome, and simultaneously providing the selective force to fix such changes, acting as drivers of bacterial evolution, with potentially adverse consequences for human welfare (Gillings, 2013). To minimize the risk of superbugs selection, the World Health Organization (WHO, 2014) recommends that hospitals have onsite facilities for the pre-treatment of hospital effluent prior to discharge into the general wastewater streaming^1^; the purpose is to eliminate different contaminants, such as bacterial pathogens, antibiotics, disinfectants, radioactive substances, toxic chemicals, etc.

Considering the increasing risk of microbial infections by reclaiming water through processing it in WWTPs, advanced treatment technologies and disinfection process are considered a major tool to control the spread of ARB into the environment (Rizzo et al., 2013), one of the most widely used and accepted ways to achieve it being through chlorine treatment (Huang et al., 2011). Chlorine efficiency depends on the concentration, exposure time and the formulation used. It has been shown that chlorine may also contribute to the selection of bacteria highly resistant to tetracycline, chloramphenicol, trimethoprim, and to the accumulation of various ARGs (such as *amp*C, *aph*A2, *bla*_TEM–__1_, *tet*A/G, *erm*A/B, plasmids, insertion sequences, and integrons) (Shi et al., 2013). In addition, metagenomic analyses performed on wastewater samples after chlorine treatment, shows that up to 40% of erythromycin resistance genes and 80% of tetracycline resistance genes cannot be removed (Yuan et al., 2015). Furthermore, chlorine treatment may promote conjugation and the ARGs transmission through horizontal gene transfer of mobile genetic elements and also plasmid over-replication and the emergence of multi-drug resistance (MDR) through activation of multi-drug efflux pumps (Shi et al., 2013; Popa et al., 2018; Sanganyado and Gwenzi, 2019; Vrancianu et al., 2020).

Multi-drug resistant *Klebsiella pneumoniae* is a major nosocomial pathogen, causing infections with high morbidity and mortality rates (up to 50%) (Bassetti et al., 2018), caused by limited treatment options. This pathogen harbors a wide resistome that could evolve under antibiotic and biocide selective pressure, leading to the occurrence of extremely drug resistant (XDR) or high-risk (HiR) clones, with great epidemic potential (Navon-Venezia et al., 2017).

As many as 90,000 infections and more than 7,000 deaths in Europe are attributable to *K. pneumoniae* resistant to carbapenems, to colistin or producing extended spectrum β-lactamase (ESBL) (Cassini et al., 2015). In 2018, resistance to carbapenems (last resort antibiotics) in *K. pneumoniae* ranged in various countries from 0 to 63.9%, the highest prevalence being encountered in Greece, followed by Romania (29.5%) and Italy (26.8%) (EARSS-Net), Europe being considered “epidemic” for carbapenemase- producing *K. pneumoniae* (Bassetti et al., 2018). The dissemination of MDR *K. pneumoniae* strains from hospitals to the environment was previously demonstrated (Mahon et al., 2017; Khan et al., 2018; Lepuschitz et al., 2019), highlighting the ability of these strains to survive and persists in environmental conditions. Despite this evidence, primary treatment of hospital wastewaters before their discharge in the urban sewage is not mandatory in many countries (Hocquet et al., 2016; Rozman et al., 2020), these wastewaters ending up being treated in urban wastewater treatment plants. One of the primary treatments of hospital wastewaters used in Romania is represented by chlorine treatment, but the knowledge regarding the effects of chlorination on ARB is scarce. The current literature reports conflicting results, since some studies describe the removal of some ARB by chlorine treatment (Zhang et al., 2015; Lin et al., 2016), while other data suggest that this treatment is ineffective (Yuan et al., 2015).

Previous data from our research team (Surleac et al., 2020) showed that MDR, carbapenemase and ESBL-producing *K. pneumoniae* isolated from clinics, hospital wastewater, and urban WWTPs in different regions of the country exhibit multiple antibiotic and antiseptic resistance, as well as virulence genes, the ST101 clone being the most frequently encountered in all sampling sites. The *K. pneumoniae* ST101 clone seems to be well established in Romanian hospitals (Dortet et al., 2015; Czobor et al., 2016) and wastewaters (Surleac et al., 2020), this determining us to investigate its possible transmission from hospital to wastewater, aiming to comparatively characterize the *K. pneumoniae* ST101 isolated from an infectious diseases hospital and its wastewaters.

## Materials and Methods

### Isolation of ARB From Clinical and Wastewater Samples

**FIGURE 1:**
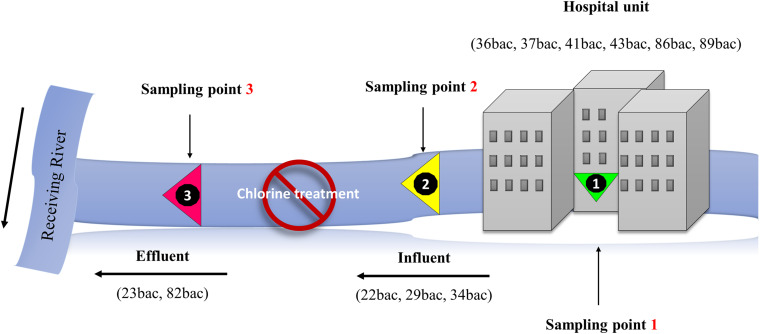
Schematic representation of the sampling sites.

Grab water samples were collected in November (21^st^ and 23^rd^) 2018, and March (20) 2019 from the influent and the effluent of the hospital collecting sewage tank (Figure 1), in which active chlorine solution (0.06 g/L) is intermittently pulverized, according to the hospital standard operating procedure. Water samples were processed following the recommendations of SR EN ISO 9308-2/2014 (coliform bacteria). Briefly, different water volumes and dilutions (undiluted 1 ml, 10 ml, and 30 ml as well as 1 ml and 3 ml out of ten-fold dilutions – 1/10 and 1/100, respectively) were filtered through 0.45 μm pore size membranes (Millipore, France), subsequently inoculated on antibiotic-enriched, chromogenic media (BioMérieux, France), namely ChromID ESBL (for ESBL-producing enterobacteria), ChromID OXA-48 agar and ChromID CARBA agar for carbapenemase (CRE)-producing strains. Up to ten colonies with KESC (*Klebsiella – Enterobacter – Serratia – Citrobacter*) carbapenem-resistance phenotype were randomly selected from each culture media per sample. The isolates were confirmed by subsequent cultivation on the same type of chromogenic media used for their isolation, identified using the MALDI-TOF-MS Bruker system and subsequently introduced in the microbial collection of the Research Institute of the University of Bucharest. In order to evaluate the occurrence of particular clones of MDR *K. pneumoniae* in hospital wastewater, 10 days prior to water sampling, all *K. pneumoniae* strains isolated from positive clinical specimens, were collected by the hospital microbiology laboratory and provided for comparative analysis (Supplementary Table 1). The clinical strains were sampled from inpatients, cultured and subsequently isolated on blood agar, and Cystine Lactose Electrolyte Deficient (CLED) agar (Rafila et al., 2015), identified using MALDI-TOF-MS Bruker system, and included in the microbial collection of the Research Institute of the University of Bucharest without any link to personal data regarding the patients.

### Antibiotic Susceptibility Testing

The two colonies of *K. pneumoniae* selected from each chromogenic media (ChromID ESBL, OXA-48, and CARBA) and each volume/dilution used for isolation, as well as the clinical *K. pneumoniae* strains were further studied using the disk diffusion method (CLSI, 2019) and the following 16 antibiotics: amikacin (AK), amoxicillin-clavulanic acid (AMC), ampicillin (AMP), aztreonam (ATM), cefepime (FEP), cefoxitin (FOX), ceftriaxone (CRO), cefuroxime (CXM), ciprofloxacin (CIP), ertapenem (ETP), gentamicin (CN), imipenem (IMP), meropenem (MEM), piperacillin (PRL), tetracycline (TET), trimethoprim-sulfamethoxazole (SXT). *Escherichia coli* ATCC 25922 strain was used for quality control.

### Screening of β-Lactam Resistance Genes (ARGs)

In order to investigate the genetic support of β-lactam enzymatic resistance (carbapenemases, ESBLs), bacterial DNA was extracted using an adapted alkaline extraction method (Almahdawy et al., 2019). The genetic background of AR was investigated by PCR (PCR Master Mix 2X, Thermo Scientific), using 1 μl of DNA and specific primers for *bla*_TEM_ (Quinteros et al., 2003), *bla*_SHV_ (Kim and Lee, 2000), *bla*_CTX–M_ (Israil et al., 2013), *bla*_OXA–__48_ (Poirel et al., 2011), *bla*_VIM_ (Shirani et al., 2016), *bla*_IMP_ (Shirani et al., 2016), *bla*_NDM_ (Nordmann et al., 2011), *bla*_SIM_ (Qi et al., 2008), *bla*_SPM_ (Ellington et al., 2007), and *bla*_KPC_ (Poirel et al., 2011) genes (Supplementary Table 2). All reactions were carried out using the temperature of 55°C for primers annealing, except for *bla*_TEM_ and *bla*_IMP_, for which the annealing was performed at 52°C.

### WGS and Bioinformatics Setup

The aquatic strains from the three sampling points (*n* = 23) showing matching AR profiles with the clinical strains (*n* = 8) (Supplementary Table 3) were selected for whole genome sequencing. Total DNA was extracted (DNeasy UltraClean Microbial Kit Qiagen optimized with an additional mechanical and chemical bacterial lysis step and ethanol precipitation) and subjected to Illumina (Nextera DNA Flex Library Prep Kit) sequencing. Both quality (2100 Bioanalyzer, Agilent) and quantity (Qubit 4 Fluorometer, Thermo Fisher Scientific, United States) checks were performed on the DNA pool libraries before starting the paired-end shotgun sequencing on the MiSeq platform (Illumina, United States). MiSeq reagent kit v.3 (600 cycles) was chosen for the high quality of the generated output.

Raw reads were quality-checked using FastQC (Andrews, 2010), assembled using *Shovill* pipeline (Seemann, 2018), and primarily annotated using Prokka (Seemann, 2014). Specific gene profiling was assessed using ABRicate (Seemann, 2020b) software, with specific databases for AR (Feldgarden et al., 2019), virulence (Chen et al., 2016), and plasmid replicons (Carattoli et al., 2014). ARIBA was also used for predicting the resistance genes for each sample (Hunt et al., 2017). Moreover, we also interrogated the database provided by the online CGE platform – http://www.genomicepidemiology.org/- (CGE Platform, 2020), regarding the resistance (Zankari et al., 2012), virulence (Joensen et al., 2014) and the pathogenic potential using Pathogen Finder predictor software (Cosentino et al., 2013). Capsular and LPS antigens (K and O loci) and chromosomal mutations involved in AR were annotated using Kleborate (Wyres et al., 2016; Wick et al., 2018). Strain relatedness was investigated using MLST (Seemann, 2020a), cgMLST (Jolley and Maiden, 2010) Snippy (Seemann, 2015) and kSNP3 (Gardner et al., 2015). Comparative gene analyses were performed using Roary (Page et al., 2015) and the output was used to infer phylogenies using RAxML (Stamatakis, 2014) and visualized using iTOL (Letunic and Bork, 2019).

### Strains Selection Based on Phenotypic and Molecular Data

Out of a total of 101 carbapenem resistant *K. pneumoniae* strains, 78 isolated from wastewater and 23 from inpatients, some of them previously characterized for their antibiotic susceptibility profiles (Surleac et al., 2020), 31 were initially selected for the present study, based on their sampling location and MDR phenotype, as defined by Magiorakos et al., 2012 (Supplementary Table 3). For all the 31 strains, the MLST profile was inferred from WGS data (Surleac et al., 2020). The MLST profiles revealed by WGS data analyses highlighted that the *K. pneumoniae* ST101 subtype was the most prevalent (*n* = 11, 36%) in all three sampling points (Supplementary Figure 2). Thus, these isolated were further selected for characterization.

## Results

For the 31 strains selected, the antibiotic susceptibility profiles, the genetic background of β-lactam resistance and ST-types were compared, (Supplementary Table 3) revealing the presence of matching patterns of AR profiles (i.e., resistance to same classes of antibiotics, or to a single antibiotic from an antibiotic class), the presence/absence of ARGs and the abundance of *K. pneumoniae* ST101 The PCR for detection of β-lactam resistance genes revealed the presence of carbapenemases genes, which were, in decreasing frequency order, *bla*_OXA–__48_, *bla*_NDM_, and *bla*_KPC_ as well as of the *bla*_CTX–M_ encoding for ESBLs. The *bla*_SHV_ gene was detected in all isolates (Supplementary Table 3).

All these strains were further characterized by whole genome sequencing. The ARGs identified by gene prediction were diverse and encoded resistance to β-lactams, aminoglycosides, quinolones, folate inhibitors, tetracyclines and others; no significant decrease of ARG distribution in the chlorine-treated effluent of vs. untreated influent of the hospital chlorination tank was observed, the majority of the ARGs being present in the aquatic strains isolated from all three sampling points. The most frequent genes encoding for β-lactam resistance were *bla*_CTX–M–__15_, *bla*_OXA–__1_, *bla*_OXA–__48_, *bla*_SHV–__106_, *bla*_TEM–__1_, and *bla*_SHV–__158_, for aminoglycosides were *aac(3)II-a*, *aph(6)-Id, aph(3*″*)-Ib*, and *aadA2*, while for quinolone resistance the transferable *qnrS1* gene was the most abundant (Supplementary Figure 1).

The main characteristics of the selected *K. pneumoniae* ST101 strains were: the presence of the MDR phenotype, three wastewater-sourced strains being resistant to all tested antibiotics (22 bac, 23 bac, 34bac), while the other two were susceptible only to aztreonam (29 bac, 82 bac). All six clinical strains were resistant to all tested antibiotics except for amikacin to which four strains were susceptible (Supplementary Table 3).

### ST101 *K. pneumoniae* Clones Carry Multiple Antibiotic Resistance, Virulence, and Biocides Resistance Determinants

All *K. pneumoniae* ST101 strains selected for WGS presented the KL17/O1v1 serotype (as predicted by Kleborate software). All of them harbored multiple common ARGs (Table 1) encoding for β-lactam (mainly carbapenemase *bla*_OXA–__48_), quinolone (*oqxA10, oqxB17*) and trimethoprim (*dfrA14*) resistance, as well as chromosomal mutations involved in resistance to quinolones (mutations of *gyrA* and *parC*), colistin (truncation of *mgrB* gene) and linked with the decreased susceptibility to cephalosporins and carbapenems (truncation of porin encoding gene *OmpK35*).

**TABLE 1:** Various features of the *K. pneumoniae* ST101 selected strains.

**Water sampling date**	**Source**	**Chromogenic media**	**Strain code**	**Common features**					**Additional features**			
				**Serotype**	**Acquired antibiotic resistance**	**Chromosomal mutations involved in antibiotic resistance**	**Virulence genes**	**Biocides resistance genes**	**Acquired antibiotic resistance**		**Chromosomal mutations involved in antibiotic resistance**	
21 Nov 2018	Influent	ChromID CARBA	29bac						*bla*_OXA–1_	*aph(6)-Id, aph(3*″*)-Ib*	*tet(D), sulII, catB3*	OmpK 36GD
			34bac									
23 Nov 2018	Influent	ChromID OXA-48	**22bac**						*bla*_OXA–1_ *bla*_CTX–M–15_ *bla*_TEM–150_	*aac (3)-IIa aac (6)-Ib-cr*	*tet(D), catB3*	–
							*entA; entB;*					
							*entE; entS;*					
							*fepA; fepB;*					
	Effluent	ChromID CARBA	**23bac**				*fepC; fepD;*					
							*fepG; fimC;*					
Nov 2018	Clinical	–	**36bac**				*fimE; fyuA;*					OmpK 36GD
			37bac		*bla*_OXA–48_,	Δ*OmpK35*,	*irp1; irp2;*		*bla*_NDM–1_	*rmtC; aph(6)-Id, aph(3*″*)-Ib*	*dfrA12, sulII*,	OmpK 36GD
					*bla*_SHV–106_		*ompA;*					
							*yagV/ecpE;*	*merA*				
			**41bac**	K17/O1v1	*dfrA14*	Δ*mgrB*,	*yagW/ecpD;*	*mdfA*		*aac (3)-IIa*	*tet(D), catB3*	
					*fosA*	GyrA-83Y	*yagX/ecpC;*					
			**43bac**		*oqxA10*	GyrA-87N	*yagY/ecpB;*			*aac (6)-Ib-cr*		OmpK 36GD
							*yagZ/ecpA;*		*bla*_OXA–1_			
20 Mar 2020	Effluent	ChromID CARBA	82bac		*oqxB17*	ParC-80I	*ybtA; ybtE;*		*bla*_CTX–M–15_ *bla*_TEM–150_	*aac (3)-IIa*		–
							*ybtP; ybtQ;*					
March 2020	Clinical	–	86bac									
							*ybtS; ybtT;*					
			89bac				*ybtU; ybtX;*			–		
							*ykgK/ecpR*					

*Bold values represents the closely related strains.*

Virulence determinants harbored by the analyzed strains were represented by siderophores, such as enterobactin (*entABCDEF*, *fepABCDG*) and yersiniabactin (*irp1, irp2, ybtSXQPA, fyuA*), as well as the operon *ecpRABCDE*, involved in adherence and biofilm formation. The biocides resistance gene *mdfA* encodes resistance to quaternary-ammonium compounds, sodium hydroxide and other biocides, while *merA* encodes resistance to mercury (Table 1).

Additionally, the majority of the strains harbored ESBL (*bla*_TEM–__150_, *bla*_CTX–M–__15_), tetracyclines – *tet(D)*, aminoglycoside- mainly *aac(3)-IIa*, chloramphenicol -*catB3* genes and presented an amino acid substitution in OmpK36 porin, which is known to be associated with carbapenem resistance (Table 1).

The pathogenic potential was estimated to 90%, according to Pathogen Finder predictor software (Cosentino et al., 2013). All strains harbored the same plasmid replicons: IncFIA, IncL/M, IncR_1, and Col, respectively.

**FIGURE 2:**
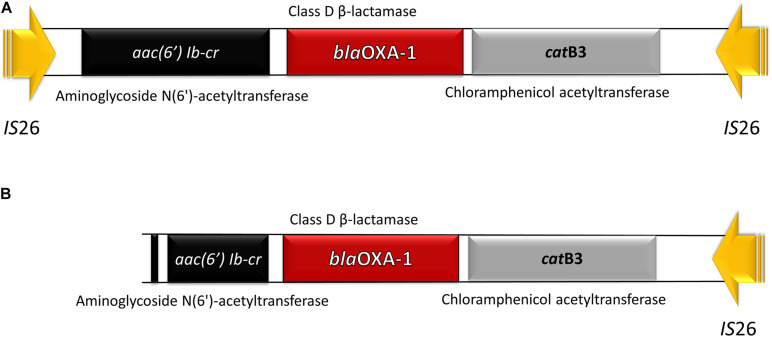
Antibiotic resistance (AR) platform carrying three different resistance genes: *aac (6*′*) lb-cr*, *bla*_OXA–1_, and *catB3*. **(A)** The whole fragment flanked by two copies of *IS*26. **(B)** Truncated *aac(6*′*) Ib-cr* gene.

The mobile genetic platform harboring *aac(6′)-Ib-cr – bla*_OXA–__1_ – *catB3* was encountered in all isolates, in three of them, respectively, the clinical strain 36bac and the wastewater isolates 34bac and 29bac being flanked by *IS*26 at both ends. In the other tested strains the *aac(6′)-Ib-cr* gene was truncated (this might be probably due to sequencing limitations) (Figure 2).

### *K. pneumoniae* ST101 Clone Survives in Hospital Chlorinated Effluent

Core SNPs analyses revealed that three clinical strains isolated in November 2018 (36bac, 41bac, and 43 bac) are closely related (≤18 SNPs) with two strains isolated on the 23^rd^ November 2018 from influent (22bac) and effluent (23bac), respectively; the wastewater sourced strains presented no SNPs in their core site (Table 2). Moreover, cgMLST profiling revealed the same allelic content. Additionally, 99% of the total genes detected in these strains were common (core-genes). This low variability allowed us to hypothesize that the three selected strains belong to the same clone, more as they were tracked in the same temporal sequence, in three spatial points of the hospital-wastewater transmission chain.

**TABLE 2:** Matrix representation of calculated SNPs distances between the closely related strains (≤18 SNPs highlighted in gray).

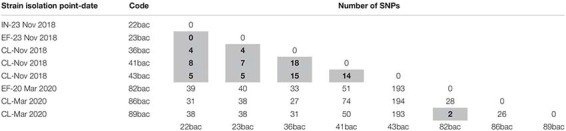

**FIGURE 3:**
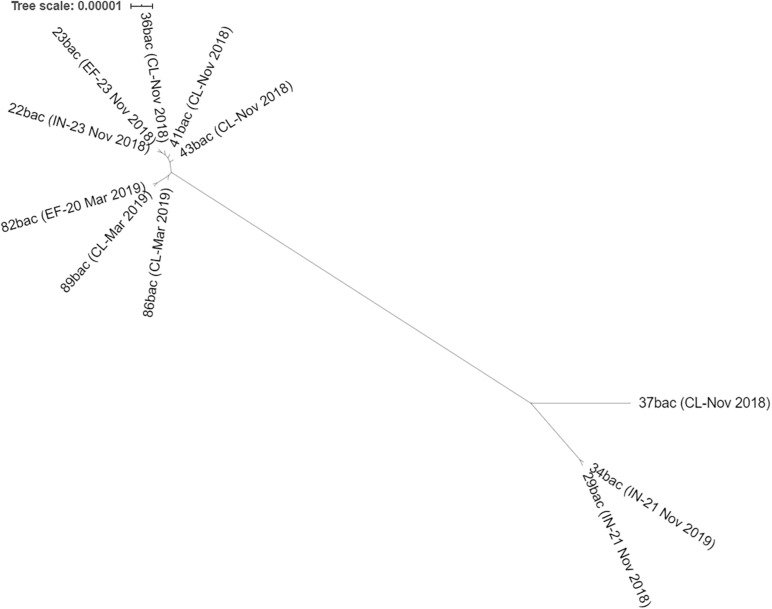
Maximum likelihood phylogeny of all ST101 *K. pneumoniae* isolated from hospital sewage: influent (IN), effluent (EF) of chlorination tank, and from clinical samples (CL) with isolation date.

These strains, isolated in November 2018, were related with those isolated in March 2019 (Table 2), where one clinical strain (89 bac) was found to be closely related with a strain isolated from the hospital effluent (82 bac) and having the same cgMLST profile. All strains presented in Table 1 are clustered together in the Maximum likelihood phylogeny (Figure 3). Two strains from influent (29 bac, 34 bac) and one clinical strain (37 bac) isolated in November 2018, although belonging to ST101 clone, were more distantly related (55–799 SNPs) and belong to a different cluster.

## Discussion

Although clinically relevant clones, such as carbapenem producing *K. pneumoniae* have been isolated in different environments, data on the occurrence and characteristics of *K. pneumoniae* resistant strains in environmental sources are still scarce (Chi et al., 2019). Our study describes the presence of the same clone of *K. pneumoniae* MDR in the hospital wastewater, in the untreated influent as well in the effluent of the chlorination tank, highlighting the inefficiency of the chlorine treatment in removing MDR *K. pneumoniae* from hospital wastewater before being released to urban wastewater collecting system.

In order to prove the clonal dissemination of *K. pneumoniae* from the clinical compartment to the hospital wastewater, a detailed characterization of the ST101 MDR *K. pneumoniae* strains recovered from hospital wastewater before and after chlorination and inpatients was performed. We have demonstrated that five MDR *K. pneumoniae* ST101 strains isolated from intra-hospital infections and two strains isolated from hospital wastewater (hospital WWTP influent and effluent) in November 2018 belong to the same clone, harboring common AR, virulence, and biocides resistance genes, two strains belonging to the same clone being isolated also in March 2019 (isolated from inpatients and from the hospital WWTP effluent, respectively).

The most important feature of the successful *K. pneumoniae* ST101 clone isolated in November 2018 was its resistance to multiple antibiotics, encoded by chromosomal mutations, as well as by resistance genes acquired through horizontal gene transfer, located within mobile genetic elements which could potentially disseminate to commensal bacterial strains. The virulence determinants harbored by this successful clone were represented by siderophores such as enterobactin (*entABCDEF*, *fepABCDG*) and yersiniabactin (*irp1, irp2, ybtSXQPA, fyuA*), their co-presence being associated with an increased risk of respiratory tract infections (Bachman et al., 2011), as well as the operon *ecpRABCDE*, involved in adherence and biofilm formation. The variants of capsular and somatic antigens KL17 and O1v1 were described as strongly associated with *K. pneumoniae* ST101 (Roe et al., 2019). In addition, the O1 antigen has been described as a major contributor to the virulence of pyogenic liver abscess causing *K. pneumoniae* (Hsieh et al., 2012). Additionally, the biocides resistance gene *mdfA* encodes resistance to quaternary-ammonium compounds, sodium hydroxide and other biocides, while *merA* encodes resistance to mercury. Although genes associated with resistance and virulence are usually identified in separate subpopulations of *K. pneumoniae* there are strains harboring both high resistance and virulence (Lam et al., 2019; Wyres et al., 2020b) making them highly pathogenic and almost impossible to treat (Wyres et al., 2020b).

Our data point that the *K. pneumoniae* clone presented here may have the ability to survive in the urban wastewater after being released in the hospital sewage. The persistence of this clone in the hospital effluent after chlorination indicates its dissemination potential in surface and recreational waters, as previously suggested by other authors (Lepuschitz et al., 2019; Suzuki et al., 2020).

Therefore, multi-level studies are needed for increasing knowledge on ecology, population structure and pathogenicity of resistant *K. pneumoniae* strains and to elucidate the possible transmission of clinical strains into the environment and the subsequent potential risk posed to human and environmental health (Wyres et al., 2020a).

Extended spectrum β-lactamase and carbapenem producing *K. pneumoniae* were previously isolated from different components of the hospital sewage treatment facilities, demonstrating the dissemination of ESBL producers between intra-hospital infections and the final effluent after the treatment process (Prado et al., 2008). The identification of VIM and KPC-producing *Klebsiella* spp. in the treated wastewater of a hospital (Gomi et al., 2018), of OXA-48 producing *K. pneumoniae* from hospital sewage (Zurfluh et al., 2017), or of the presence of the same clone in the hospital sewage and receiving river (Suzuki et al., 2020) raises concerns. Since its first detection in Székely et al. (2013), OXA-48 was the most frequently encountered carbapenemase in Romania (Lixandru et al., 2015; Grundmann et al., 2017; Popescu et al., 2017; Baicus et al., 2018; Surleac et al., 2020).

The IncFII, IncN, IncR, and IncX3 incompatibility groups are most often associated with the horizontal gene transfer of AR genes in *K. pneumoniae* determined mainly by the presence of the following replicons: FIB_K_, FII_K_, R, Col, FII, FIA (all identified in this study) FIB, X, N, HI1B, AC/2 (Wyres et al., 2020a). Beside these replicons, we have also identified the IncL/M replicon in all isolates. The acquired ARGs in *K. pneumoniae* ST101 isolates are associated with particular mobile elements, e.g., the association of *bla*_CTX–M–__15_ with IncFII plasmids while *bla*_OXA–__48_ was often identified in IncL/M plasmids (Wyres and Holt, 2016), as also revealed by our results.

One might consider a limitation of this study the low number of the selected MDR *K. pneumoniae* strains belonging to the same clone traveling from hospital to WWTP effluents. Further studies, with more sampling campaigns and more isolates are required to provide the epidemiological link from the clinical compartment to the water bodies through hospital wastewater as well as the correlation between the persistence of the clones and different exposure times to chlorination treatment.

## Conclusion

The survival of ARB in treated hospital wastewater is very alarming and highlights the necessity of an improved surveillance and the need to elucidate the role of the environment in the transmission and dissemination of MDR *K. pneumoniae* strains. The isolation of the same clone from both hospital and WWTP influent and effluent after chlorination suggests the highly adaptive potential of the clone and highlights the need for further studies designed to track the fate of these clones after release from hospital in the aquatic environment. In addition, disinfection strategies for hospital wastewaters should be reconsidered, in the light of such novel epidemiological data.

## Data Availability Statement

The datasets presented in this study can be found in online repositories. The names of the repository/repositories and accession number(s) can be found below: https://www.ncbi.nlm.nih.gov/, BioProject PRJNA579879.

## Author Contributions

LP: conceptualization, data curation, methodology, software, formal analysis, and writing – original draft. IG: conceptualization, data curation, methodology, formal analysis, and writing – original draft. IB: conceptualization, data curation, formal analysis, software, and writing – original draft. MS: data curation, formal analysis, software, and writing – review and editing. SP: conceptualization, methodology, and writing – review and editing. LM, MP, GP, DT, MN, and AnS-C: methodology. AdS-C: conceptualization. DO: conceptualization and writing – review and editing. MC: conceptualization, data curation, formal analysis, funding acquisition project administration, supervision, and writing – review and editing. All authors contributed to the article and approved the submitted version.

## Conflict of Interest

The authors declare that the research was conducted in the absence of any commercial or financial relationships that could be construed as a potential conflict of interest.

## Footnotes

1 https://www.who.int/

## Supplementary Material

Supplementary Figure 1 The distribution of antibiotic resistance (AR) genes to β-lactam antibiotics. **(A)** aminoglycosides and quinolones, **(B)** and other classes of antibiotics, **(C)** predicted from WGS data in the analyzed *K. pneumoniae* strains.

Supplementary Figure 2 MLST profiles of the *K. pneumoniae* strains isolated from the clinical compartment, influent, and effluent of the chlorination tank.
