# Supplementary figures and images for: Multidrug Resistant Klebsiella pneumoniae ST101 Clone Survival Chain From Inpatients to Hospital Effluent After Chlorine Treatment
Source: Front Microbiol. 2021 Jan 11;11:610296. doi: 10.3389/fmicb.2020.610296 (PMC7873994; doi:10.3389/fmicb.2020.610296)

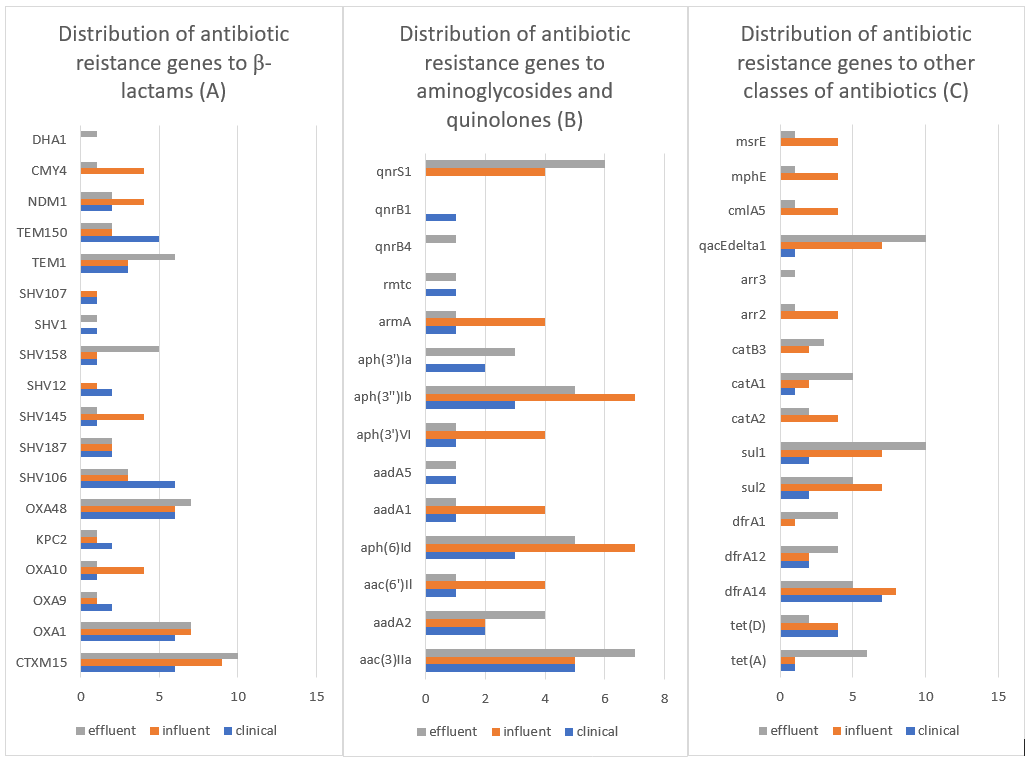

Supplement: Supplementary Figure 1 — The distribution of antibiotic resistance (AR) genes to β-lactam antibiotics. (A) aminoglycosides and quinolones, (B) and other classes of antibiotics, (C) predicted from WGS data in the analyzed K. pneumoniae strains. [file Image_1.TIFF]

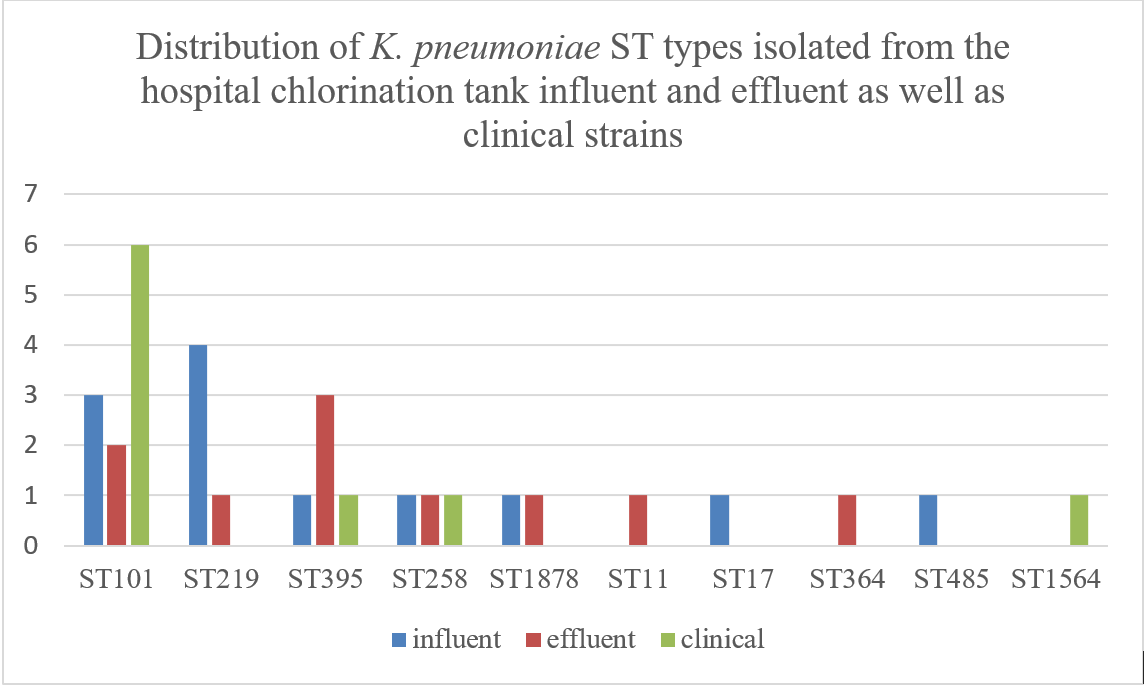

Supplement: Supplementary Figure 2 — MLST profiles of the K. pneumoniae strains isolated from the clinical compartment, influent, and effluent of the chlorination tank. [file Image_2.TIFF]
