# Supplementary material for: Multidrug Resistant Klebsiella pneumoniae ST101 Clone Survival Chain From Inpatients to Hospital Effluent After Chlorine Treatment
Source: Front Microbiol. 2021 Jan 11;11:610296. doi: 10.3389/fmicb.2020.610296 (PMC7873994; doi:10.3389/fmicb.2020.610296)
Supplement: Supplementary file 3 [file Table_1.DOCX]

| Isolation point  (total no. of isolated *K. pneumoniae*) | Influent  (n=42) | Effluent  (n=36) | Clinical  (n=23) |
| --- | --- | --- | --- |
| **Total number of *K. pneumoniae* isolated in each campaign** | 21 Nov 2018 – n=18  23 Nov 2019 – n=18 | 21 Nov 2018 – n=12  23 Nov 2018 – n=18 | n=14 |
|  | 20 March 2019 – n=6 | 20 March 2019 – n=6 | n=9 |

Supplementary table 1. The distribution of carbapenem resistant *K. pneumoniae* isolates used as a basis for the selection of the *K. pneumoniae* strains deeply analyzed in this study
