# Supplementary material for: Multidrug Resistant Klebsiella pneumoniae ST101 Clone Survival Chain From Inpatients to Hospital Effluent After Chlorine Treatment
Source: Front Microbiol. 2021 Jan 11;11:610296. doi: 10.3389/fmicb.2020.610296 (PMC7873994; doi:10.3389/fmicb.2020.610296)
Supplement: Supplementary file 4 [file Table_2.DOCX]

Supplementary table 2. Primer sequences used in the characterization of samples and PCR amplicon sizes

| **Gene** | **Primer sequences** | **Amplicon**  **size**  **(bp)** | **Reference** |
| --- | --- | --- | --- |
| ***bla*_TEM_** | TEM-F: ATAAAATTCTTGAAGACGAAA  TEM-R: GTCAGTTACCAATGCTTAATC | 1080 | Quinteros et al. 2003 |
| ***bla*_SHV_** | SHV-F: TGGTTATGCGTTATATTCGCC  SHV-R: GGTTAGCGTTGCCAGTGCT | 870 | Kim and Lee, 2000 |
| ***bla*_CTX-M_** | CTX-M-F: CGCTGTTGTTAGGAAGTGTG  CTX-M-R: GGCTGGGTGAAGTAAGTGAC | 754 | Israil et al., 2013 |
| ***bla*_OXA-48_** | OXA-48-F: GCGTGGTTAAGGATGAACAC  OXA-48-R: CATCAAGTTCAACCCAACCG | 438 | Poirel et al., 2011 |
| ***bla*_VIM_** | VIM-F: GATGGTGTTTGGTCGCATA  VIM-R: CGAATGCGCAGCACCAG | 390 | Shirani et al., 2016 |
| ***bla*_IMP_** | IMP-F: GGAATAGAGTGGCTTAA(C/T)TCTC  IMP-R: GGTTTAA(C/T)AAAACAACCACC | 232 | Shirani et al., 2016 |
| ***bla*_NDM_** | NDM-F: GGTTTGGCGATCTGGTTTTC  NDM-R: CGGAATGGCTCATCACGATC | 621 | Nordmann et al., 2011 |
| ***bla*_SIM_** | SIM-F: TACAAGGGATTCGGCATCG  SIM-R: TAATGGCCTGTTCCCATGTG | 570 | Qi et al., 2008 |
| ***bla*_SPM_** | SPM-F: AAAATCTGGGTACGCAAACG  SPM-R: ACATTATCCGCTGGAACAGG | 271 | Ellington et al., 2007 |
| ***bla*_KPC_** | KPC-F: CGTCTAGTTCTGCTGTCTTG  KPC-R: CTTGTCATCCTTGTTAGGCG | 798 | Poirel et al., 2011 |
