# Supplementary material for: Multidrug Resistant Klebsiella pneumoniae ST101 Clone Survival Chain From Inpatients to Hospital Effluent After Chlorine Treatment
Source: Front Microbiol. 2021 Jan 11;11:610296. doi: 10.3389/fmicb.2020.610296 (PMC7873994; doi:10.3389/fmicb.2020.610296)
Supplement: Supplementary file 5 [file Table_3.DOCX]

Supplementary Table 3. Antibiotic susceptibility testing results and the genetic background of β-lactam resistance for the analysed *K. pneumoniae* strains (the ST101 strains selected for further investigations are highlighted in gray)

| **Sampling point** | **Sampling date** | **Strain ID** | **Antibiotic Susceptibility** | | | | | | | | | | | | | | | | | | | **ARG identified by PCR** | **MLST profile** |
| --- | --- | --- | --- | --- | --- | --- | --- | --- | --- | --- | --- | --- | --- | --- | --- | --- | --- | --- | --- | --- | --- | --- | --- |
|  |  |  | **AMP** | **PRL** | **AMC** | **CXM** | **FOX** | **CRO** | **CTX** | **KZ** | **FEP** | **ETP** | **IMP** | **MEM** | **ATM** | **AK** | **CN** | **TET** | **CIP** | **SXT** | **MDR** |  |  |
| Hospital chlorination tank  **influent** (*n* = 12) | 21.11.2018 | **29bac** | R | R | R | R | R | R | - | - | R | R | R | R | S | R | R | R | R | R | + | OXA-48, SHV | ST 101 |
|  |  | **34bac** | R | R | R | R | R | R | - | - | R | R | R | R | R | R | R | R | R | R | + | OXA-48, SHV | ST 101 |
|  |  | **35bac** | R | R | R | R | R | R | - | - | R | R | R | R | R | R | R | R | R | R | + | CTX-M, NDM, SHV | ST 219 |
|  |  | **24bac** | R | R | R | R | S | R | - | - | R | R | S | R | R | S | R | S | R | R | + | CTX-M, SHV, TEM | ST 485 |
|  | 23.11.2018 | **21bac** | R | R | R | R | R | R | - | - | R | R | R | R | R | R | R | R | R | R | + | CTX-M, NDM, OXA-48, SHV, TEM | ST 219 |
|  |  | **22bac** | R | R | R | R | R | R | - | - | R | R | R | R | R | R | R | R | R | R | + | CTX-M, OXA-48, SHV, TEM | ST 101 |
|  |  | **30bac** | R | R | R | R | R | R | - | - | R | R | R | R | R | R | R | S | R | R | + | CTX-M, NDM, SHV | ST 219 |
|  |  | **31bac** | R | R | R | R | R | R | - | - | R | R | R | R | R | R | R | S | R | R | + | CTX-M, NDM, SHV | ST 219 |
|  |  | **32bac** | R | R | R | - | S | R | - | - | R | R | R | R | R | S | R | S | S | S | - | CTX-M, SHV | ST 17 |
|  | 20.03.2019 | **68bac** | R | R | S | R | S | - | R | R | R | S | S | S | R | S | - | S | S | S | - | CTX-M, SHV | ST 1878 |
|  |  | **69bac** | R | R | R | R | R | - | R | R | R | R | R | R | R | R | - | R | R | R | + | CTX-M, OXA-48, SHV, TEM | ST 395 |
|  |  | **74bac** | R | R | R | R | R | - | R | R | R | R | R | R | R | R | - | S | R | R | + | KPC, TEM, SHV | ST 258 |
| Hospital chlorination tank  **effluent (*n* = 11)** | 21.11.2018 | **19bac** | R | R | R | R | R | R | - | - | R | R | R | R | R | R | R | S | R | R | + | CTX-M, NDM, SHV | ST 219 |
|  |  | **20bac** | R | R | R | - | S | R | - | - | R | R | S | S | R | S | S | S | S | R | - | CTX-M, OXA-48, SHV, TEM | ST 11 |
|  | 23.11.2018 | **23bac** | R | R | R | R | R | R | - | - | R | R | R | R | R | R | R | R | R | R | + | CTX-M, OXA-48, SHV, TEM | ST 101 |
|  |  | **26bac** | R | R | R | R | S | S | - | - | S | R | R | R | S | S | R | R | R | R | + | NDM-1, OXA-48, SHV | ST 101 |
|  |  | **28bac** | R | - | R | R | S | R | - | - | R | R | S | S | R | R | S | S | R | R | + | CTX-M, SHV | ST 364 |
|  | 20.03.2019 | **76bac** | R | R | R | R | R | - | R | R | R | R | R | R | R | R | - | S | R | R | + | KPC, SHV, TEM | ST 258 |
|  |  | **77bac** | R | R | S | R | S | - | R | R | R | S | S | S | R | S | - | S | S | R | - | CTX-M, SHV | ST 1878 |
|  |  | **78bac** | R | R | R | R | R | - | R | R | R | R | R | R | R | R | - | R | R | R | + | CTX-M, OXA-48, SHV, TEM | ST 395 |
|  |  | **79bac** | R | R | R | R | R | - | R | R | R | R | R | R | R | R | - | R | R | R | + | CTX-M, OXA-48, SHV, TEM | ST 395 |
|  |  | **80bac** | R | R | R | R | R | - | R | R | R | R | R | R | S | R | - | R | R | R | + | CTX-M, OXA-48, SHV, TEM | ST 395 |
|  |  | **82bac** | R | R | R | R | R | - | R | R | R | R | R | R | S | R | - | R | R | R | + | CTX-M, OXA-48, SHV, TEM | ST 101 |
| **Clinical**  **(*n* = 8)** | 11.2018 | **36bac** | R | R | R | R | R | R | - | - | R | R | S | S | R | R | R | R | R | R | + | CTX-M, OXA-48, SHV, TEM | ST 101 |
|  |  | **37bac** | R | R | S | R | S | R | - | - | R | R | R | R | R | R | R | R | R | R | + | NDM, OXA-48, SHV | ST 101 |
|  |  | **38bac** | R | R | S | R | S | R | - | - | R | S | S | S | R | R | R | R | R | R | + | CTX-M, SHV, TEM | ST 1564 |
|  |  | **41bac** | R | R | R | R | R | R | - | R | R | R | R | R | R | S | R | R | R | R | + | CTX-M, OXA-48, SHV, TEM | ST 101 |
|  |  | **43bac** | R | R | R | R | R | R | - | R | R | R | R | R | R | S | R | R | R | R | + | CTX-M, OXA-48, SHV, TEM | ST 101 |
|  | 03.2019 | **86bac** | R | R | R | R | R | - | R | R | R | R | R | R | R | S | - | R | R | R | + | CTX-M, OXA-48, SHV, TEM | ST 101 |
|  |  | **87bac** | R | R | R | R | R | - | R | R | R | R | R | R | R | R | - | S | R | R | + | KPC, SHV, TEM | ST 258 |
|  |  | **89bac** | R | R | R | R | R | - | R | R | R | R | R | R | R | S | - | R | R | R | + | CTX-M, OXA-48, SHV, TEM | ST 101 |

AMP = ampicillin, PRL = piperacillin, AMC = amoxicillin-clavulanic acid, CXM = cefuroxime, FOX = cefoxitin, CRO = ceftriaxone, CTX = cefotaxime, KZ = cefazolin, FEP = cefepime, ETP = ertapenem, IMP = imipenem, MEM = meropenem, ATM = aztreonam, CN = gentamicin, AK = amikacin, TET = tetracycline, CIP = ciprofloxacin, SXT = trimethoprim-sulfamethoxazole; MDR = multidrug resistant
